# Additive Manufacturing of Polyamide-6 Preforms for Scalable Thermal Drawing of Structured Fibers

**DOI:** 10.3390/mi17080978

**Published:** 2026-08-19

**Authors:** Akila Bandara, Ahmed Moustafa Abd-El Nabi, Luka Morita, Dan Sameoto

**Affiliations:** Department of Mechanical Engineering, University of Alberta, Edmonton, AB T6G 2R3, Canada; yapamudi@ualberta.ca (A.B.); aabdelr1@ualberta.ca (A.M.A.-E.N.); lmorita@ualberta.ca (L.M.)

**Keywords:** thermal drawing, additive manufacturing, 3D printing, Fused Deposition Modeling, FDM, polyamide-6, PA6, soft robotics, smart fabrics, smart textiles, smart fibers

## Abstract

Thermal drawing is a prominent, scalable method for transforming preforms with complex macroscopic morphologies into multifunctional microscopic fibers. With the intent to develop fibers with complex cross-sections for potential integration in soft robotics, smart textile and fabric applications, we explore the potential of polyamide-6 (PA6) as a base material for thermal drawing. By implementing Fused Deposition Modeling (FDM), we additively manufactured PA6 preforms with solid circular and three-channel cross-sectional architectures. These preforms were then utilized in a series of thermal drawing experiments to identify the best-performing printing layer thickness and channel aspect ratio (AR) that consistently yielded scalable, microscopic fiber diameters over extended durations. Our results demonstrate that a printing layer thickness of 0.3 mm yielded the most consistent drawn fiber diameters for extended durations among the tested values. Additionally, AR experiments indicate that the intermediate ARs of 0.4 and 0.5 between the inner channel diameter and the outer diameter produce the most promising results within the investigated range, based on dimensional trends and qualitative observations of internal channel integrity.

## 1. Introduction

Flexible and wearable smart sensor and actuator fibers have garnered considerable interest due to their adaptability in many human-centric applications, including human sensing, body-adaptive health monitoring, and bionic systems [1,2,3,4]. To fulfill their intended functionalities, wearable smart fibers require a higher degree of complexity, in both material composition and structural design [5,6]. Structural complexity remains a parallel focus, with some of the literature exploring complex internal geometries to enhance functional performance [2,7,8,9,10,11].

Polymer-based fibers such as polylactic acid (PLA), polyethylene (PE), and polystyrene (PS) have been co-drawn using a single thermal drawing system to produce solid fibers for sensor applications [12]. Similarly, gallium–indium alloy (EGaIn) has been integrated alongside elastomeric fibers to manufacture strain fiber sensors [13]. Graphene nanoplates (GPs) have been incorporated into polyurethane (PU) fibers for the development of temperature, resistive strain, and humidity sensors [14].

Among engineering thermoplastics, PA6 is widely utilized owing to its strong intermolecular hydrogen-bonded network, which imparts high tensile strength, exceptional toughness, and superior wear resistance [15]. These robust mechanical traits make PA6 an ideal candidate for high-performance applications, including structural micro-fibers, smart textiles, and wearable electronics [15]. However, realizing the full functional potential of PA6 in advanced fiber architectures requires precise control over cross-sectional geometry during processing.

However, finding a manufacturing technology capable of producing PA6-based fibers with complex geometries remains a significant challenge. Several studies have attempted to manufacture PA6 at the fiber scale. While electrospinning [16] and multistage drawing [17] have been utilized to process PA6 at the fiber scale, traditional melt spinning remains the dominant industrial route [16]. A major limitation of conventional spinning is that it is inherently constrained by die geometry, making it unsuitable for fabricating fibers with the complex internal features necessary for specialized functionality [18,19,20]. Alternatively, thermal drawing enables the creation of intricate, multi-material geometries [2,7,8,9,10,11,21]. By heating and stretching a macroscopic preform that contains the target cross-section, thermal drawing scales the preform down to microscopic resolution, while maintaining precise geometric proportions [11].

To date, several preform fabrication methods have been established to provide a diverse array of functional fiber architectures, including extrusion [22], 3D printing [23], direct assembly [24], thin-film rolling [25], rod-in-tube [26], deep-hole drilling [27], casting [28], and the double-crucible method [29]. Among these, 3D printing offers distinct advantages that position it at the forefront of preform manufacturing technologies. Primarily, it allows for the rapid fabrication of complex structures, and the integration of intricate geometries and materials without any requirement for prior molds or extensive preparation [11,23,30]. However, combining 3D printing with thermal drawing necessitates rigorous optimization to achieve uniform diameters while preserving delicate cross-sectional structures in micro-scale fibers. This process optimization requires fine-tuning in both 3D printing parameters and the subsequent thermal drawing conditions. Currently, a limited number of papers have discussed the impact of printing layer thickness on the consistency of drawn fiber diameter. Demircali et al. [21] investigated this relationship, demonstrating that an increased layer thickness reduces fiber diameter variation. However, their study did not evaluate this effect across different material systems. Furthermore, their work identified geometric instability during thermal drawing as a primary limitation, emphasizing the need to assess preform quality across diverse polymers and to understand how cross-sectional geometric ARs influence the dimensional accuracy of the final fibers.

To address these gaps, this study systematically aims to investigate the thermal drawability of PA6 preforms across standard solid designs and a novel three-channel geometry. Specifically, we quantify the impact of the preform’s 3D printing layer thickness on fiber diameter stability while evaluating how channel ARs govern the outer diameter consistency and the retention of internal channels.

## 2. Materials and Methods

### 2.1. Material, Preform Fabrication, and 3D Printing Parameters

**Figure 1:**
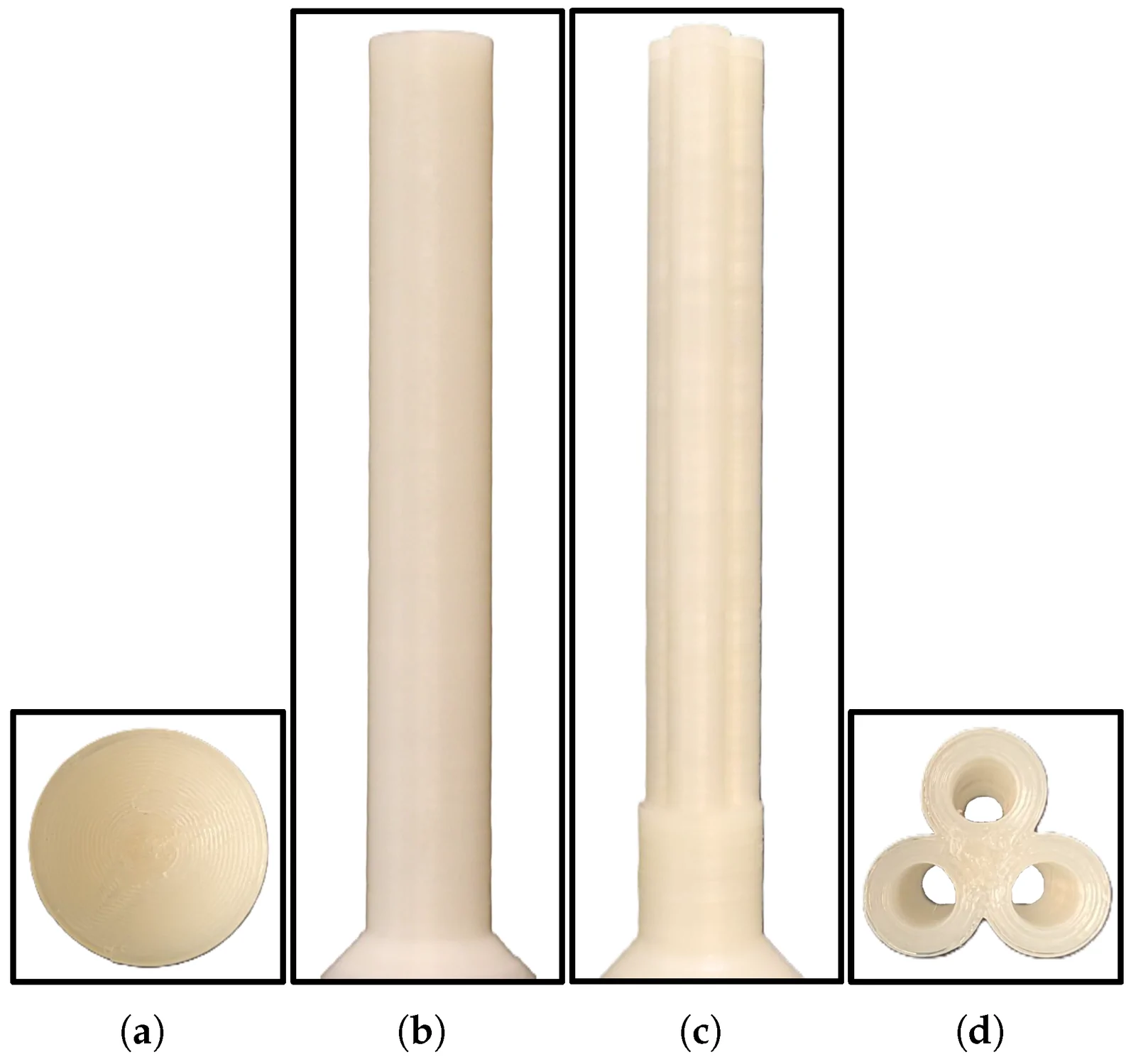
Images of the actual preforms used in experiments. Panels (**a**,**b**) illustrate the cross-sectional view and the side view of solid cylindrical preform used in layer thickness experiments. Panels (**c**,**d**) illustrate the cross-sectional view and the side view of the three-channel preform used in AR experiments.

**Figure 2:**
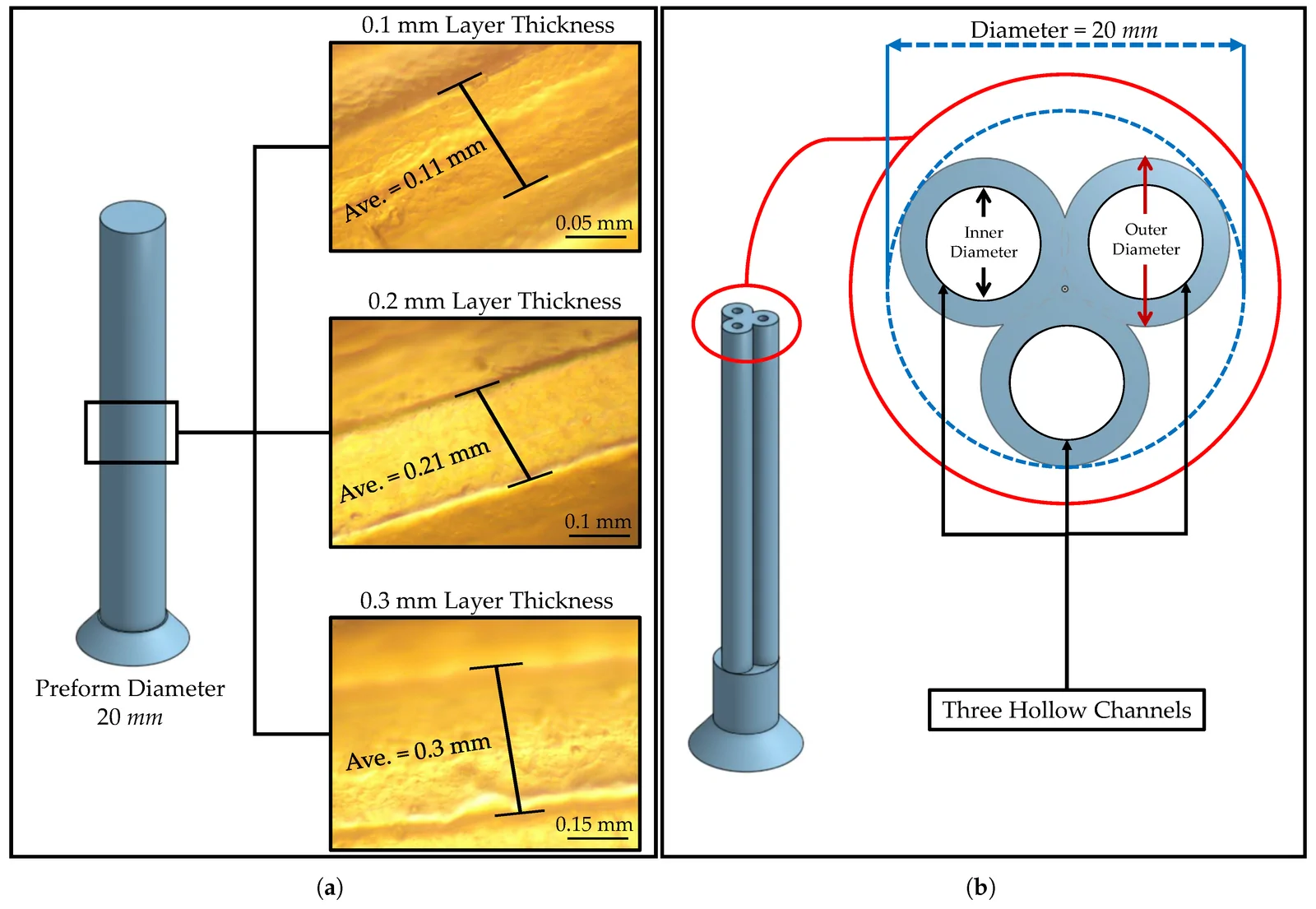
Schematics of the different preform geometries used in the experiments. Panels (**a**,**b**) are schematics of the solid cylindrical and three-channel preforms, respectively.

PA6 in pellet form, supplied by BASF SE (product: Ultramid B40 LN, ID no. 30240045, Shanghai, China), was used in this study to investigate its thermal drawability. To mitigate the highly hygroscopic nature of PA6, strict moisture control was implemented throughout all fabrication steps. Prior to the start of the extrusion process, the raw pellets were dried at 80 °C for at least 12 h. The resulting extruded filament was stored in a dehydrator at 80 °C immediately post-production and continuously between subsequent processing steps. Once 3D-printed, all preforms were again stored in a dehumidifier for conditioning at 80 °C for at least 12 h before being implemented in thermal drawing experiments [31]. The preform manufacturing process commenced with fabrication of a custom PA6 filament with a diameter of 1.55 ± 0.05 mm. A Wellzoom B-pro filament extruder featuring a 3.00 mm die diameter operating at an extrusion temperature of 265 °C was employed to extrude the PA6 pellets. A Filabot Airpath system, operating at 55% fan speed, was utilized to cool the filament along the extrusion path. A Filabot Spooler precision filament winder paired with a Filabot Filameasure was used to monitor and control the winding process. The extruded filaments were then fed into a non-enclosed SOVOL SV08 Fused Deposition Modeling (FDM) 3D printer. Solid cylindrical and three-channel preform designs were created using Onshape, a cloud-based computer-aided design (CAD) platform, and the resulting models were sliced with OrcaSlicer software. The 3D printing process parameters are as shown in Table 1. To determine the printing layer thickness that yields the most consistent thermally drawn fiber diameters over extended durations, different layer thickness values (0.1 mm, 0.2 mm, and 0.3 mm) were assessed by 3D printing four solid cylindrical preforms for each layer thickness. Additionally, three-channel preforms, with varying ARs, as presented in Table 2, were printed and tested to evaluate the integrity of hollow channels alongside the drawn fiber’s diameter/length dimension consistency. Figure 1 illustrates images of the actual 3D-printed preforms. Figure 2 illustrates the geometric shape and dimensions of the solid cylindrical and three-channel preforms. Figure 2a shows the different layer thickness values used for printing the solid cylindrical preform. Figure 2b shows the cross-sectional dimensions that were used to calculate the ARs using Equation (1).

(1) $$\mathrm{Aspect}\;\mathrm{Ratio}\;\left(\mathrm{AR}\right)\;=\;\frac{\mathrm{Inner}\;\mathrm{Channel}\;\mathrm{Diameter}}{\mathrm{Outer}\;\mathrm{Diameter}},$$

#### 2.1.1. Thermal Drawing Process

**Figure 3:**
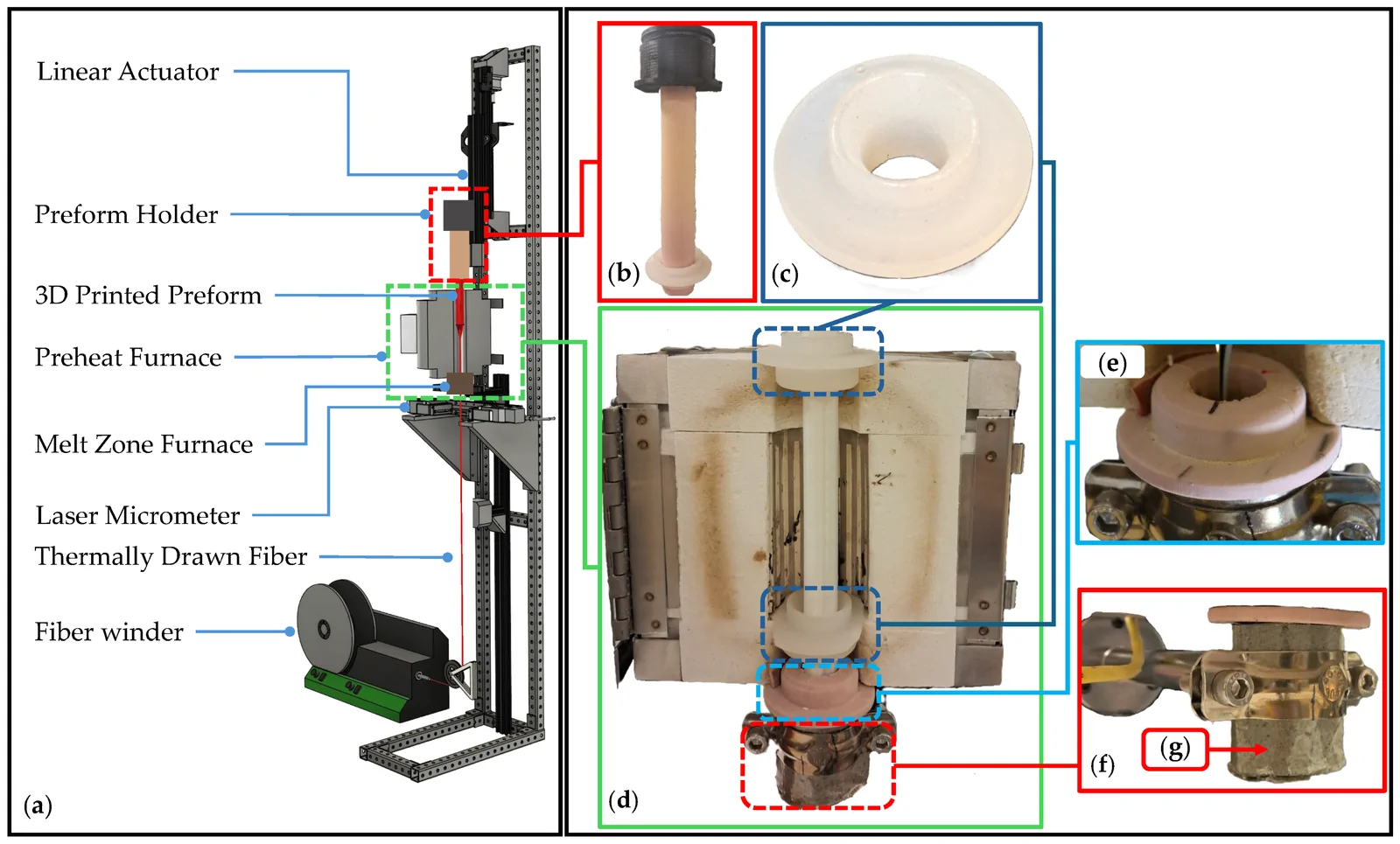
The upgraded components of the thermal draw tower. Panel (**a**) is a schematic image of the upgraded thermal draw tower. Panel (**b**) shows a closeup image of the preform holder setup. Panel (**c**) is a regular silicone rubber gasket. Panel (**d**) shows components of the two heating zones. Panels (**e**,**f**) are closeup images of a heat-resistant silicone rubber gasket and the melt zone furnace, respectively. Panel (**g**) shows the mica sheet wrapped around the melt zone furnace.

A custom-built thermal drawing tower, initially developed in a previous study [32], was implemented in this work after several design upgrades to enhance the quality of the drawn fiber. The experimental setup was upgraded by introducing a secondary heating furnace (35 mm diameter × 35 mm length) to act as a melt zone furnace, while repurposing the original furnace described in Morita et al. [32] as a preheating furnace. The connection between the preheating and the melt zone furnaces was made via a heat-resistant silicone rubber (BJB TC-5045)-cast gasket which also aligned the two furnaces. Two additional silicone rubber gaskets (Dragon Skin 30) were installed at the inlet and the outlet of the preheating furnace to further enhance alignment of the preform inside the heating zones. To improve process efficiency, the preform feeding mechanism was also upgraded, and a special preform design holder was cast from heat-resistant silicone rubber. This holder secures the preform inside the preheating and melt zone furnaces, allowing us to utilize an additional 20 cm of the preform that remained unused inside the previous furnace design [32]. The upgraded thermal draw tower is depicted in Figure 3.

To ensure that the channels remain intact during the drawing process [33], the open ends of the three-channel preforms were properly sealed with 3D-printed circular disks (20 mm in diameter and 1 mm in thickness) to trap atmospheric air inside the channels. These disks were printed using a commercial PA6 filament (Spectrum Group). Since the commercial PA6 variant has a higher melting point than the custom PA6 used to print our preforms, the seals remained solid during drawing, effectively preventing trapped air from escaping the channels. Each preform was subjected to a 10 min preheating phase at 85 °C [34], marginally above the glass transition temperature of PA6 [21], to mitigate the internal stresses accumulated in the preform throughout its production thermal history via stress relaxation phenomena [35]. The preform was then introduced into the main furnace for drawing at 270 °C (drawing temperature). To prevent thermal shock to the preform, during preheating, the melt zone furnace was kept at a temperature almost equivalent to the glass transition temperature of PA6. Eventually, heating of the melt zone furnace to 270 °C commenced once the preform was preheated and introduced into the melt zone.

The drawn fiber dimensions for the solid cylindrical and three-channel preforms were regulated by maintaining constant feeding and drawing speeds of 1.20 mm min^−1^ and 3.00 m min^−1^, respectively. The predicted value of fiber diameter/length dimension was determined using the draw ratio in Equation (2) [2]:

(2) $$r_{f}=r_{p}\sqrt{\frac{v_{f}}{v_{d}}},$$

where $r_{f}$ is fiber diameter, $r_{p}$ is the preform diameter, $v_{f}$ is the feed speed, and $v_{d}$ is the draw speed.

#### 2.1.2. Fiber Diameter Measurement

**Figure 4:**
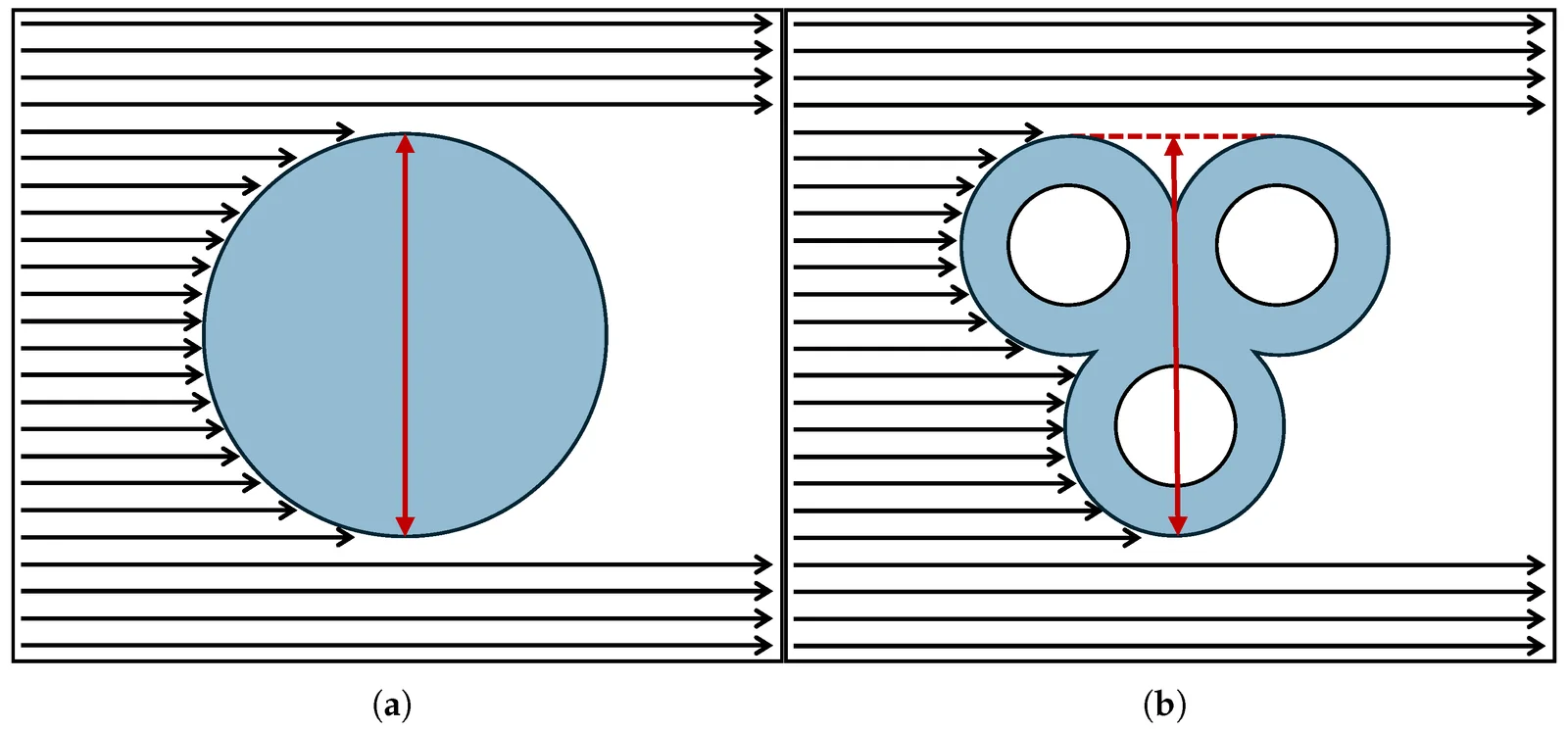
Digital representation of how the diameter/length is measured for different cross-sections of drawn fibers. Panels (**a**,**b**) are the cross-sections of the solid cylindrical and three-channel preforms, respectively. The red arrow in panel (**a**) represents the diameter measurement of the solid cylindrical fiber with a predicted theoretical value of 400 μm. The red arrow in panel (**b**) represents the measured length dimension of the three-channel fiber with a predicted theoretical value of 347.5 μm.

During the layer thickness experiments, the diameters of the solid cylindrical preform were monitored in real time using a laser micrometer (LS-9030MT, KEYENCE, Osaka, Japan) in a single direction as shown in Figure 4a. For AR experiments, the length dimension of each three-channel preform was measured by fixing the orientation of the preform, as shown in Figure 4b, inside the preheating zone and the melt zone furnace. The laser micrometer measured the drawn fiber diameter/length dimension in the direction of the red arrow shown in Figure 4a,b. The black arrows denote the optical path of the laser micrometer’s high-intensity LEDs.

Standard statistical metrics such as mean (μ), standard deviation (SD), coefficient of variation (CV), and root mean squared error (RMSE) were implemented to quantitatively compare thermal draw stability across preform layer thicknesses and ARs. For each layer thickness, RMSE values were calculated relative to the predicted theoretical fiber diameter of 400 μm. Similarly, for each AR, the RMSE values were calculated relative to the predicted theoretical length dimension of 347.5 μm illustrated in Figure 4b.

## 3. Results

This section presents results from two independent sets of experiments conducted using the thermal draw tower. First, a solid cylindrical preform architecture, 3D-printed with three different layer thicknesses, was utilized to evaluate how printing layer thickness impacted the consistency of thermally drawn fiber diameters. Second, a three-channel preform architecture was used to evaluate how internal-to-outer diameter AR impacted the consistency and the integrity of thermally drawn fibers diameters and their internal channels.

### 3.1. Layer Thickness

**Figure 5:**
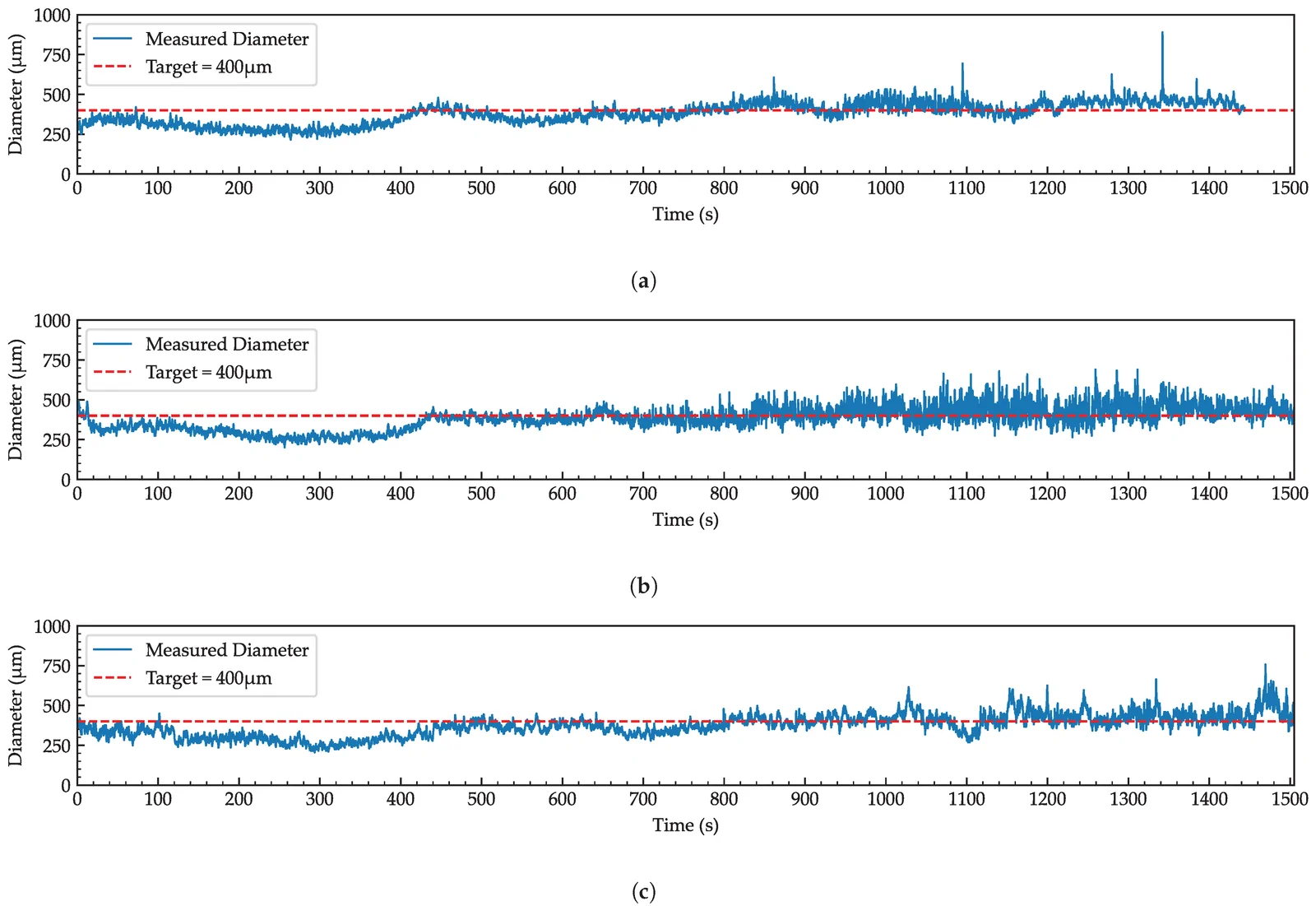

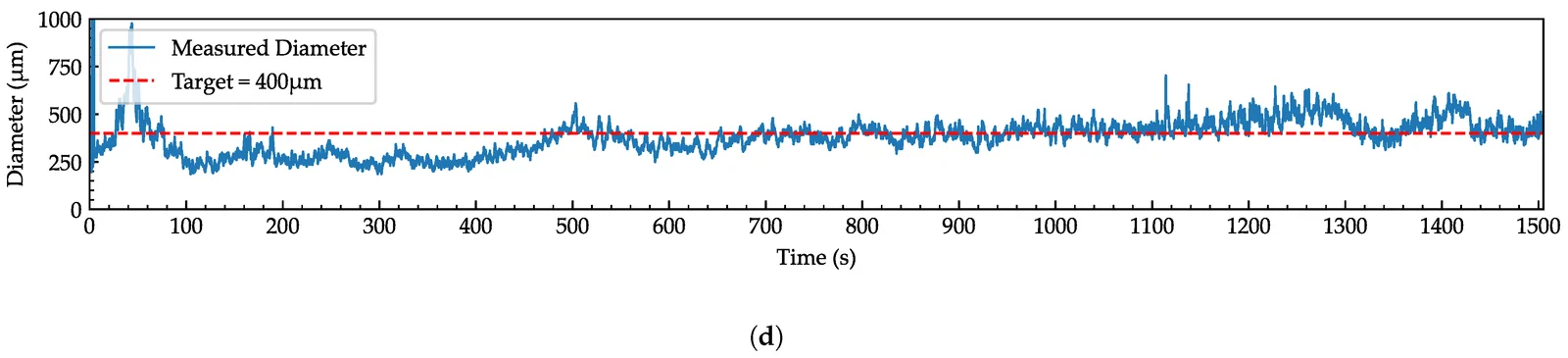
The evolution of fiber diameter vs. time of a PA6 solid cylindrical preform for a printing layer thickness of 0.1 mm. Panels (**a**–**d**) are replicates 1, 2, 3, and 4, respectively.

**Figure 6:**
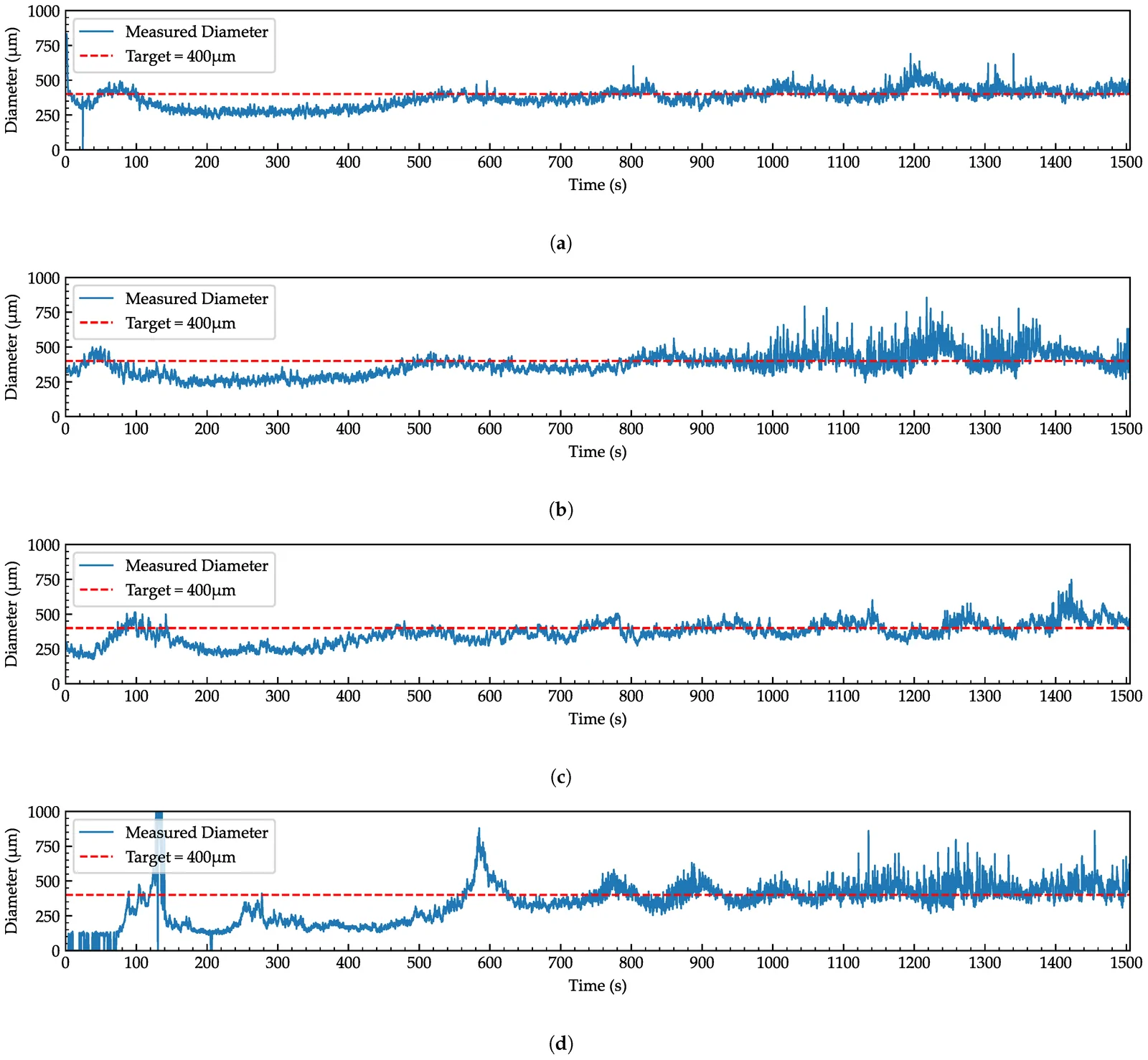
The evolution of fiber diameter vs. time of a PA6 solid cylindrical preform for a printing layer thickness of 0.2 mm. Panels (**a**–**d**) are replicates 1, 2, 3, and 4, respectively.

**Figure 7:**
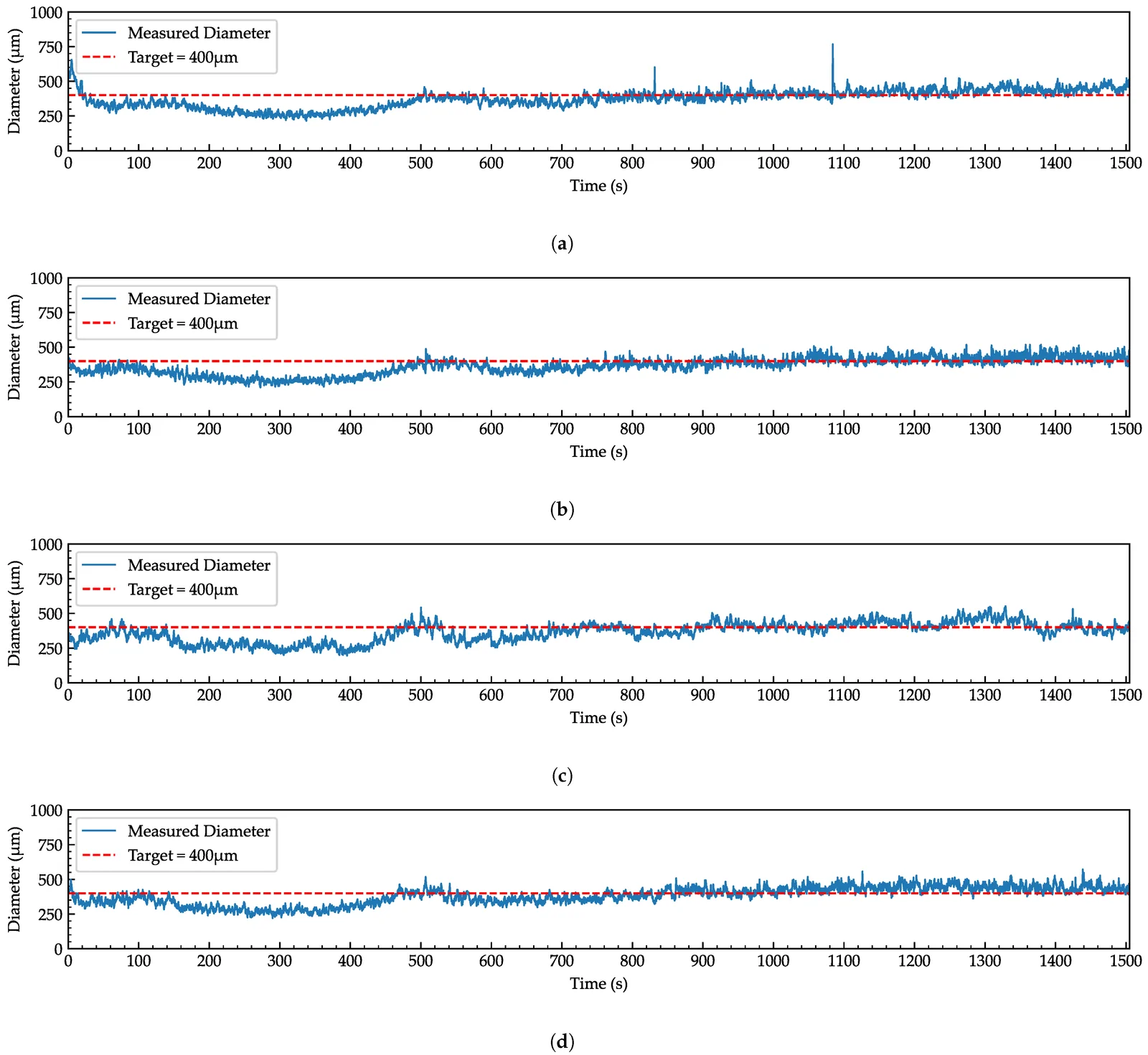
The evolution of fiber diameter vs. time of a PA6 solid cylindrical preform for a printing layer thickness of 0.3 mm. Panels (**a**–**d**) are replicates 1, 2, 3, and 4, respectively.

Figure 5, Figure 6 and Figure 7 illustrate the diameter evolution over time during the thermal drawing of PA6 solid cylindrical preforms with layer thicknesses of 0.1 mm, 0.2 mm, and 0.3 mm, respectively. Each layer thickness was evaluated based on results from four replicates. Across all layer thickness values, the measured diameters evolved transiently to reach an eventual steady state between 300 s and 700 s. In line with previous studies, this transient regime is likely governed by the initial time required to stabilize the softening of the preform [36], while reaching draw tension equilibrium [37]. Consequently, the metrics used to evaluate the best-performing layer thickness among the tested values were computed based on the diameter data for the time duration from 700 s to 1500 s (Table 3).

Based on visual inspections alone, a printing layer thickness of 0.3 mm appeared to produce the most consistent fiber diameters from PA6 solid cylindrical preforms. To quantify the observed visual distinction, each replicate was grouped by its layer thickness, and a one-way analysis of variance (ANOVA) was conducted. Since CV measures the relative variability in the fiber diameter and RMSE measures the deviation of the fiber diameter from its predicted theoretical value, separate ANOVAs were conducted for each metric. As shown in Table 4, printing layer thickness had a statistically significant impact on the consistency of fiber diameter.

To identify specific differences between individual settings, post hoc independent-sample *t*-tests were conducted on all three pairwise layer thickness combinations (0.3 mm and 0.1 mm, 0.3 mm and 0.2 mm, and 0.1 mm and 0.2 mm) for both metrics, as listed below in Table 5. The *t*-tests confirmed that CV and RMSE values of 0.3 mm layer thickness were significantly lower (i.e., superior) compared to those for the 0.1 mm and 0.2 mm layer thicknesses.

These results indicated that among the tested values, the 0.3 mm layer thickness yielded the best-performing PA6 preforms for thermal drawing of fibers.

### 3.2. Aspect Ratio

**Figure 8:**
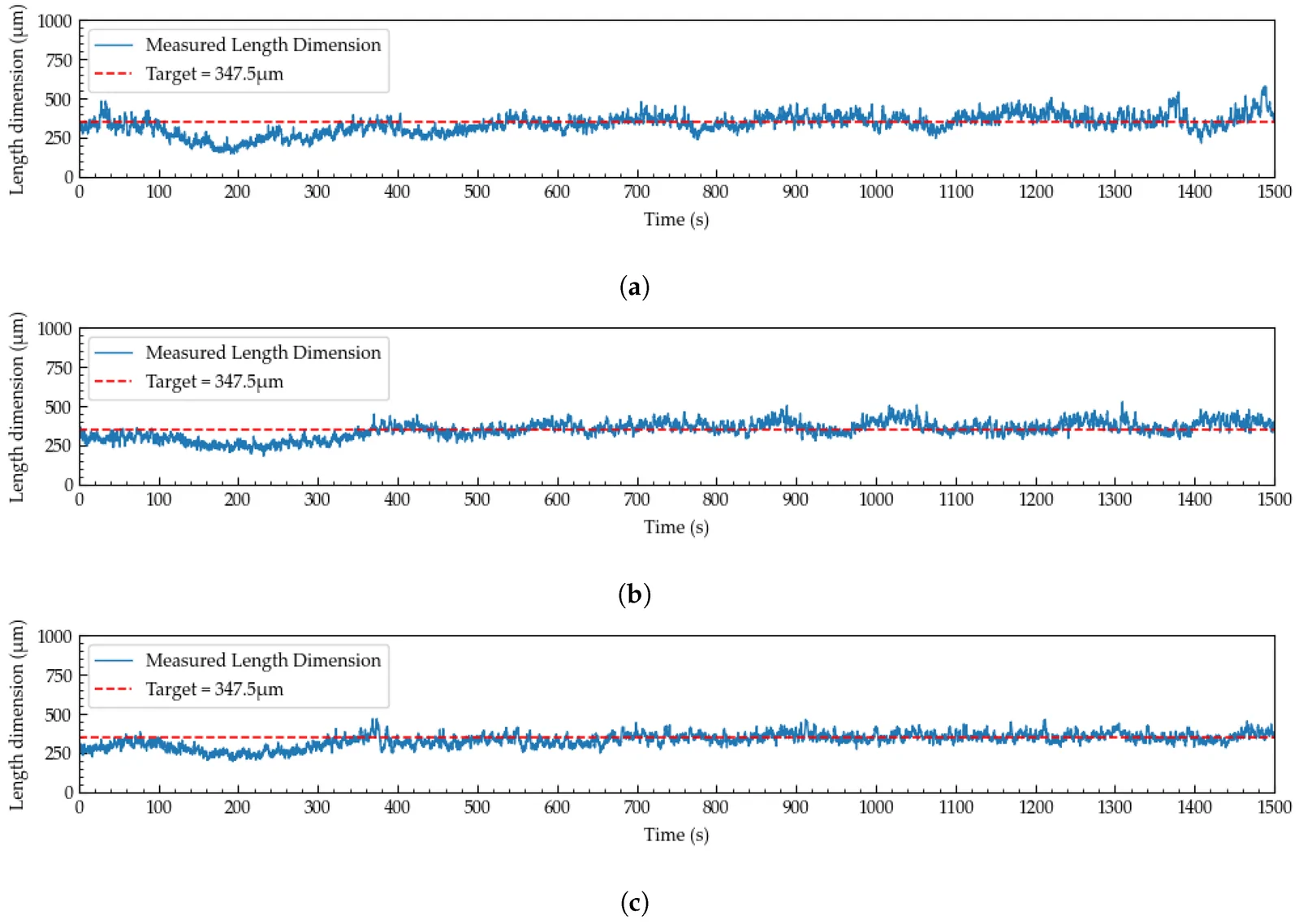
Evolution of the length dimension over time of the 1st AR (0.3) three-channel preform. Graphs in panels (**a**–**c**) show the evolution of length dimension for replicates 1, 2, and 3, respectively.

**Figure 9:**
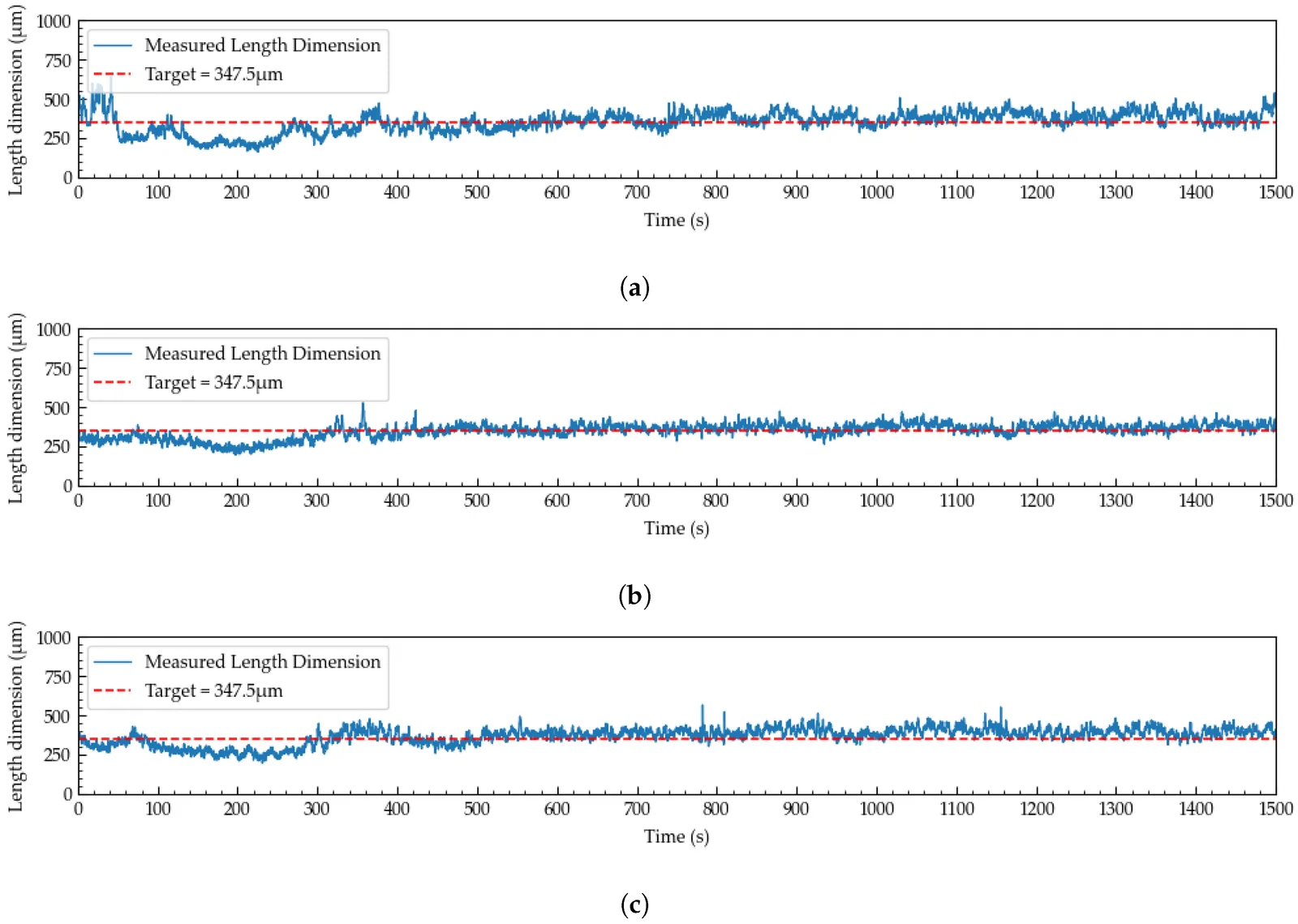
Evolution of the length dimension over time of the 2nd AR (0.4) three-channel preform. Graphs in panels (**a**–**c**) show the evolution of length dimension for replicates 1, 2, and 3, respectively.

**Figure 10:**
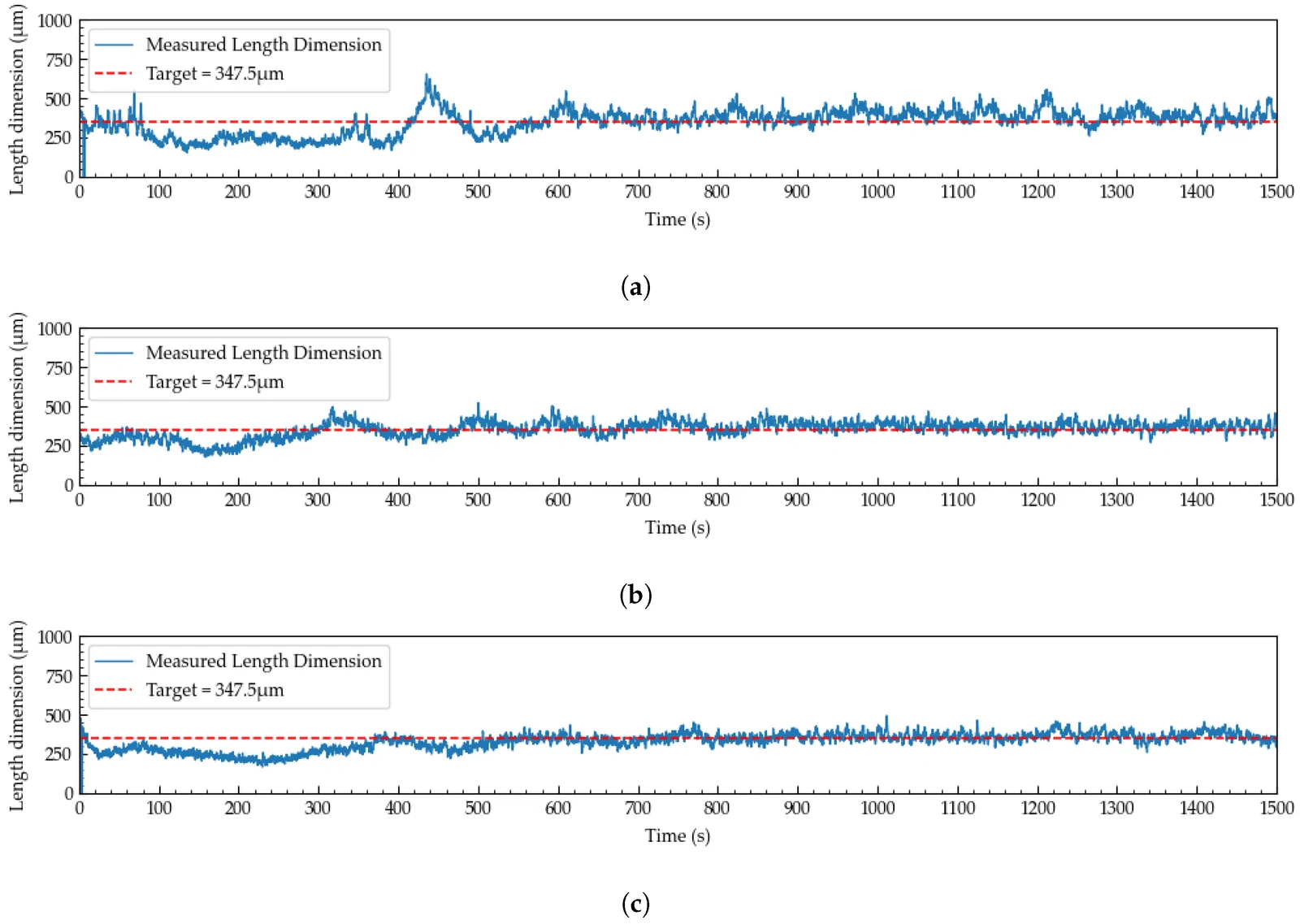
Evolution of the length dimension over time of the 3rd AR (0.5) three-channel preform. Graphs in panels (**a**–**c**) show the evolution of length dimension for replicates 1, 2, and 3, respectively.

**Figure 11:**
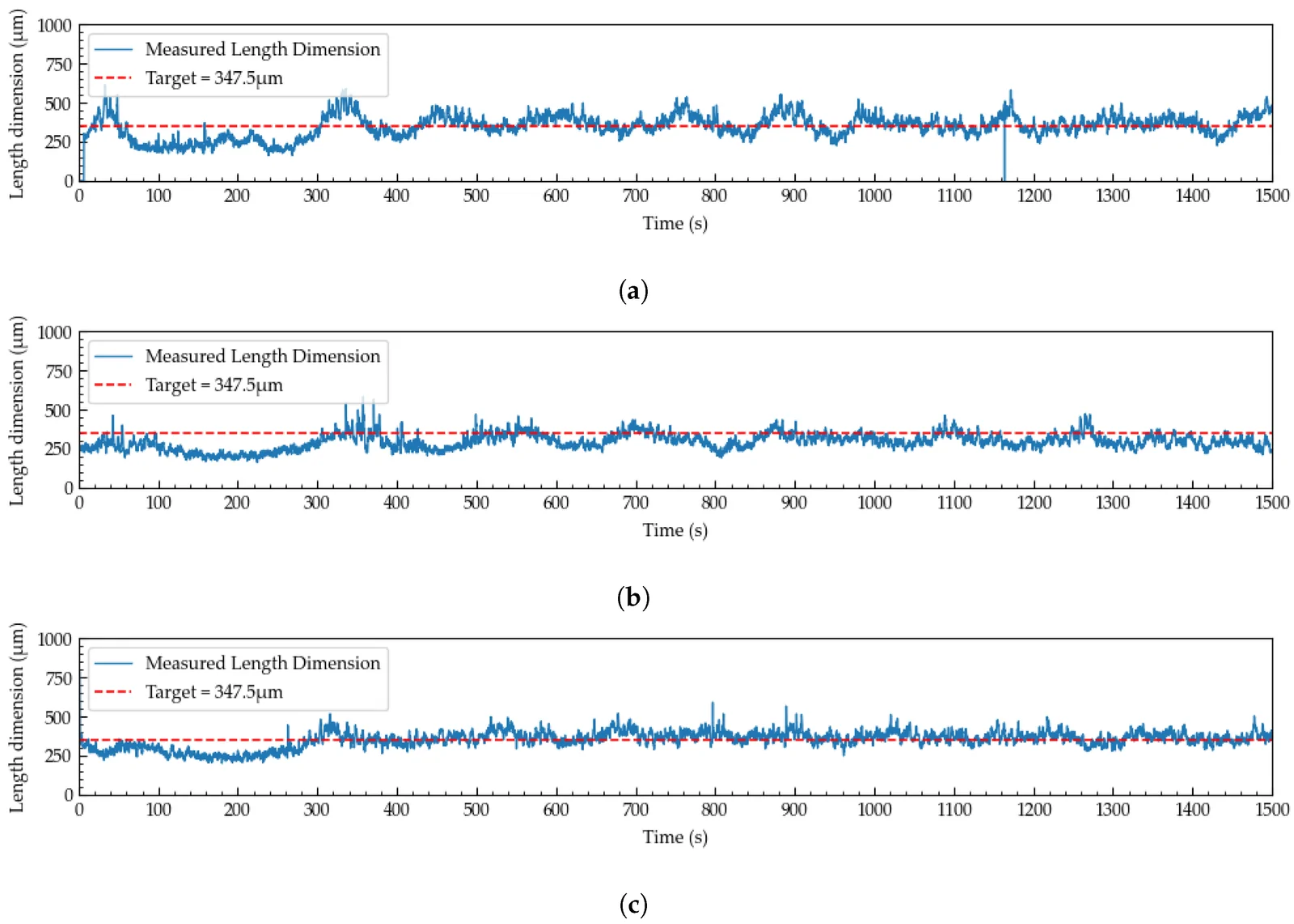
Evolution of the length dimension over time of the 4th AR (0.6) three-channel preform. Graphs in panels (**a**–**c**) show the evolution of length dimension for replicates 1, 2, and 3, respectively.

**Figure 12:**
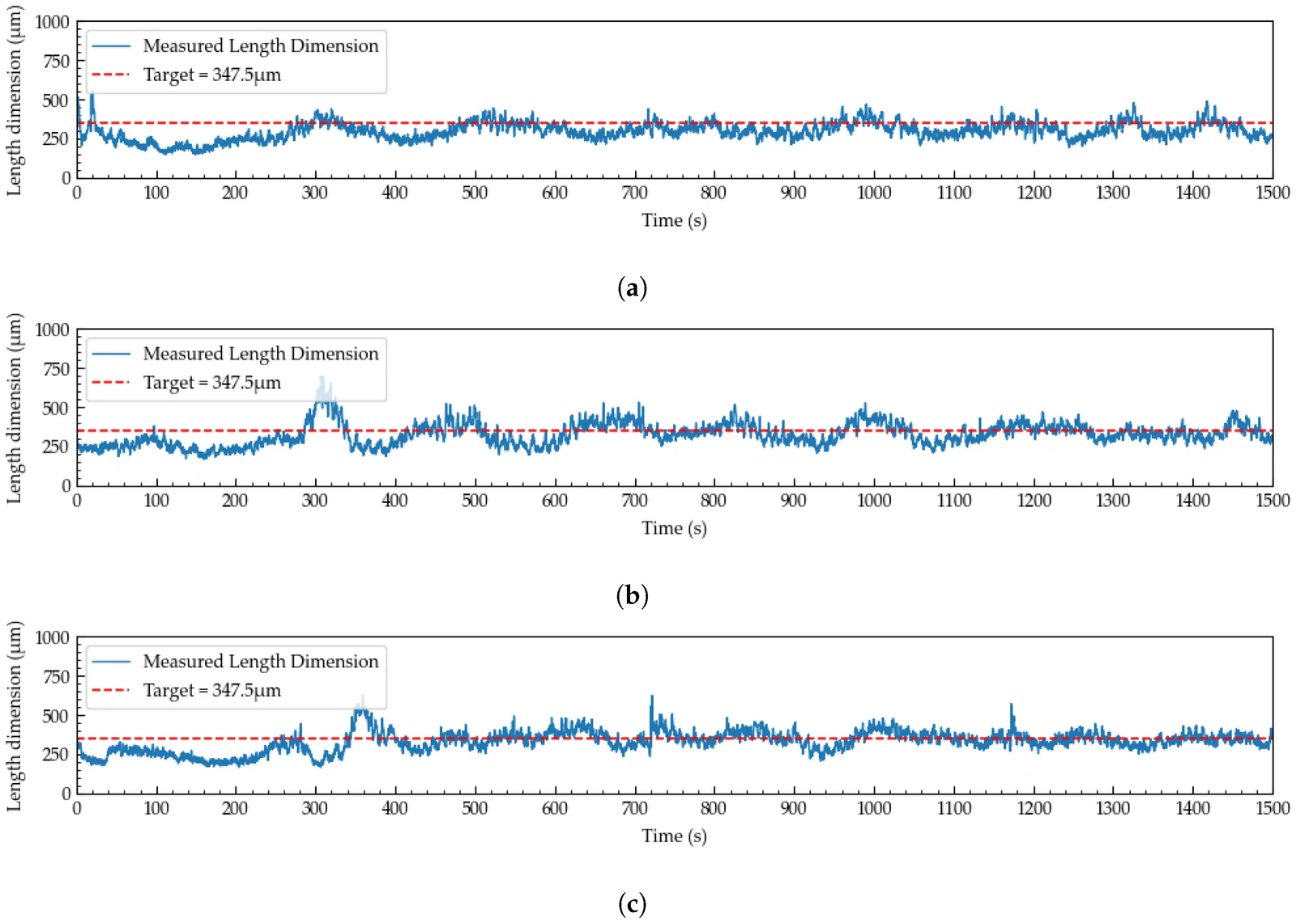
Evolution of the length dimension over time of the 5th AR (0.7) three-channel preform. Graphs in panels (**a**–**c**) show the evolution of length dimension for replicates 1, 2, and 3, respectively.

Figure 8, Figure 9, Figure 10, Figure 11 and Figure 12 illustrate the length dimension evolution over time for thermally drawn PA6 three-channel preforms with 1st (0.3), 2nd (0.4), 3rd (0.5), 4th (0.6), and 5th (0.7) ARs, respectively. Each AR was evaluated based on results from three replicates. Across all ARs, the diameters evolved transiently to reach the steady state between 300 s and 700 s. Therefore, the metrics used to evaluate the most suitable AR within the investigated range were calculated from the diameter evolution for the time duration from 700 s to 1500 s (Table 6).

One-way ANOVA was conducted independently for CV and RMSE metrics by grouping the replicates according to their ARs. As shown in Table 7 statistically significant difference across ARs was observed only for CV values.

To determine specific differences between individual settings, post hoc Holm-adjusted pairwise comparisons were conducted on all 10 pairwise AR combinations for the CV metric, as listed below in Table 8.

**Figure 13:**
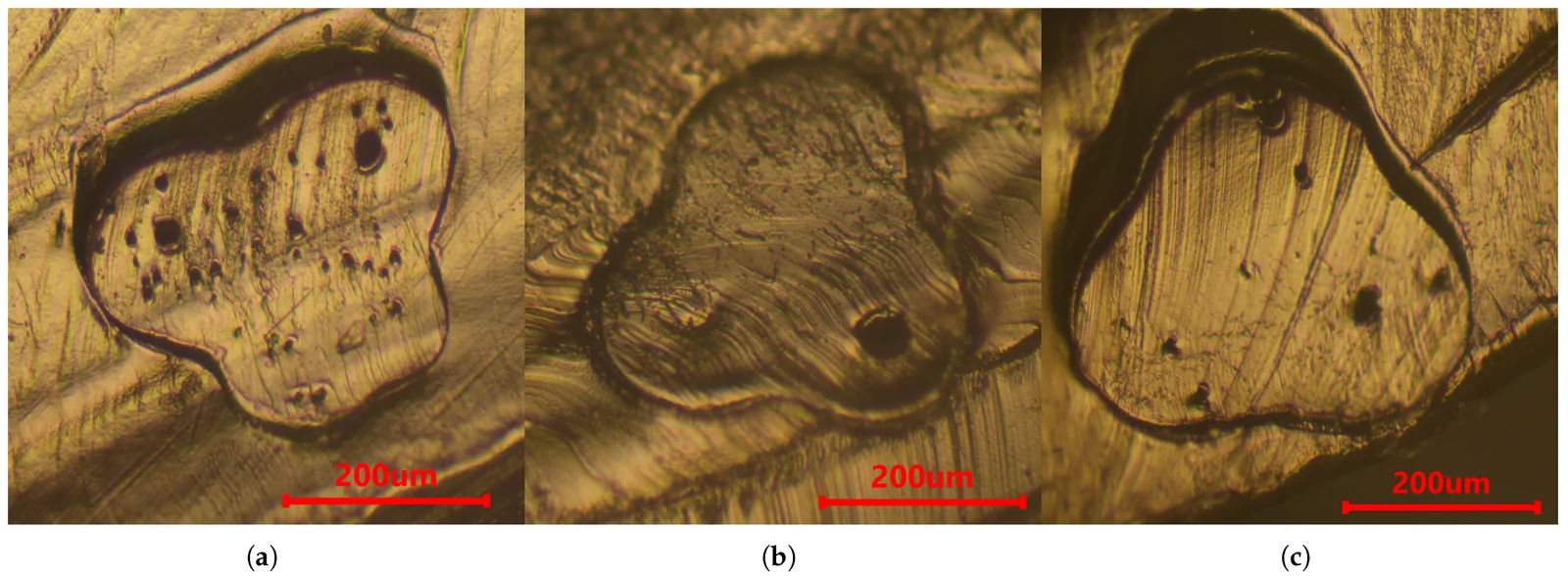
Microscopic images of drawn fiber cross-sections of PA6 three-channel preforms. Panels (**a**–**c**) shows the cross-sections of replicates 1, 2, and 3 of the 1st (0.3) AR, respectively.

**Figure 14:**
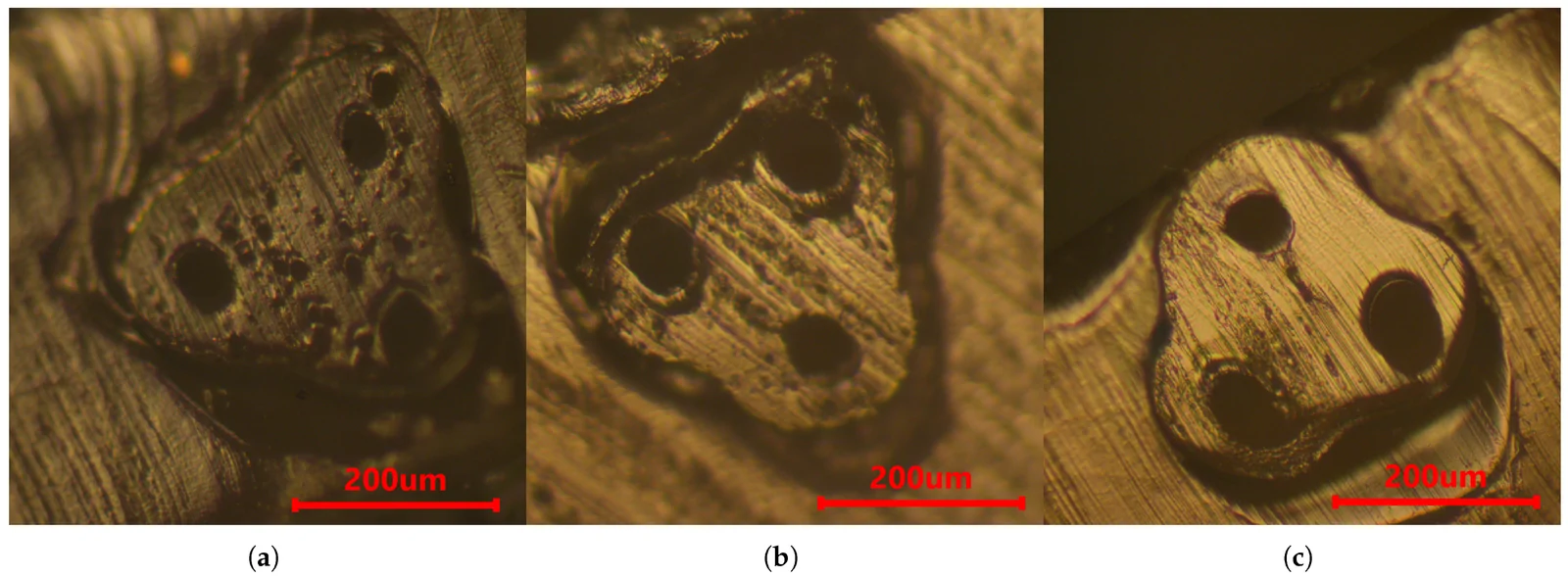
Microscopic images of drawn fiber cross-sections of PA6 three-channel preforms. Panels (**a**–**c**) show the cross-sections of replicates 1, 2, and 3 of the 2nd (0.4) AR, respectively.

**Figure 15:**
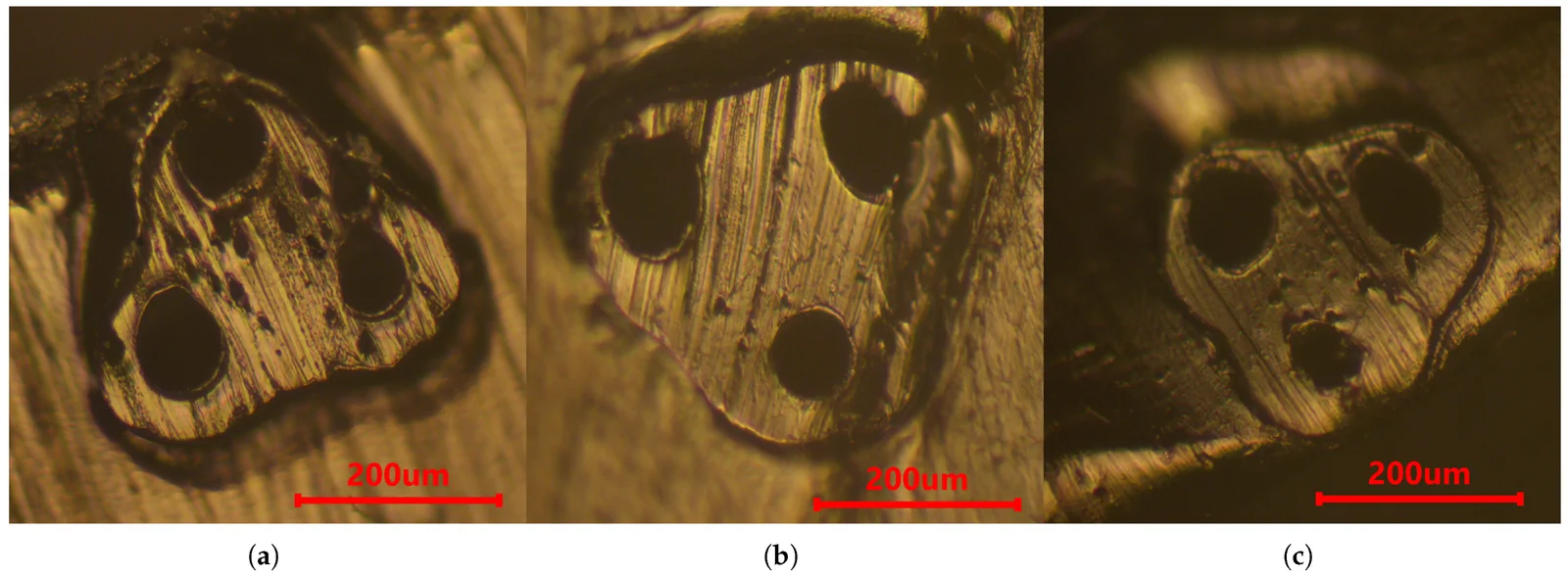
Microscopic images of drawn fiber cross-sections of PA6 three-channel preforms. Panels (**a**–**c**) show the cross-sections of replicates 1, 2, and 3 of the 3rd (0.5) AR, respectively.

**Figure 16:**
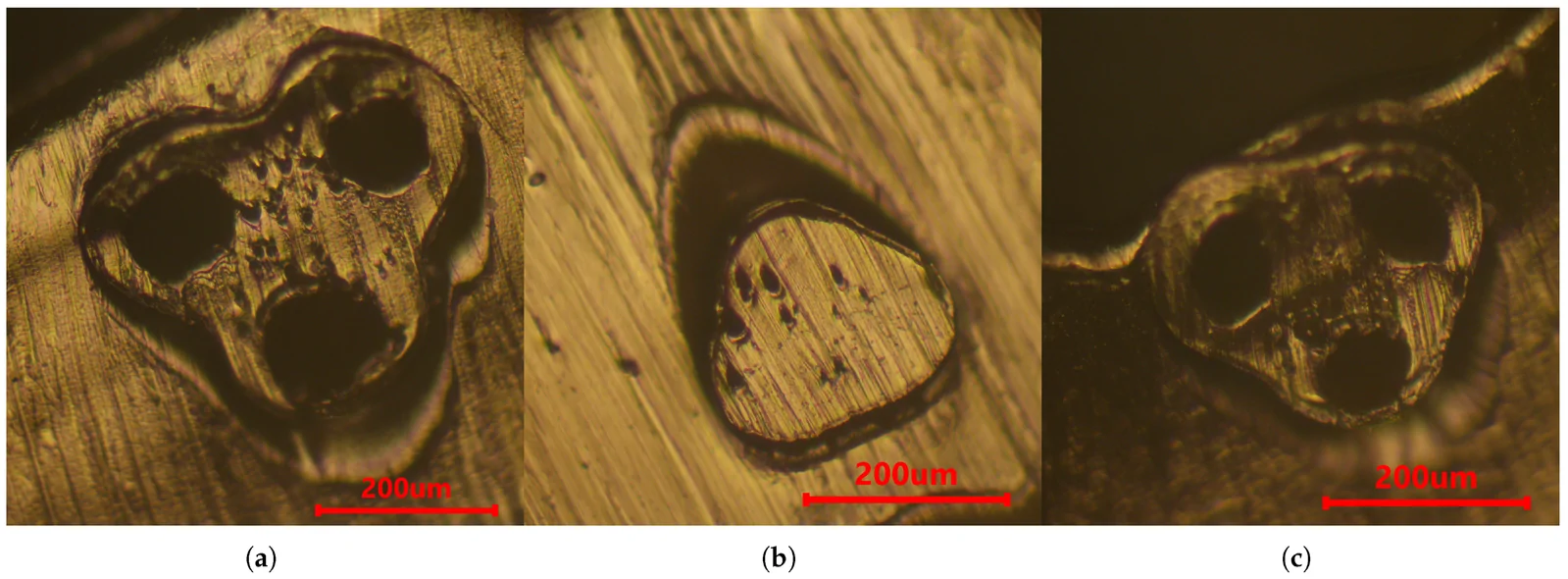
Microscopic images of drawn fiber cross-sections of PA6 three-channel preforms. Panels (**a**–**c**) show the cross-sections of replicates 1, 2, and 3 of the 4th (0.6) AR, respectively.

**Figure 17:**
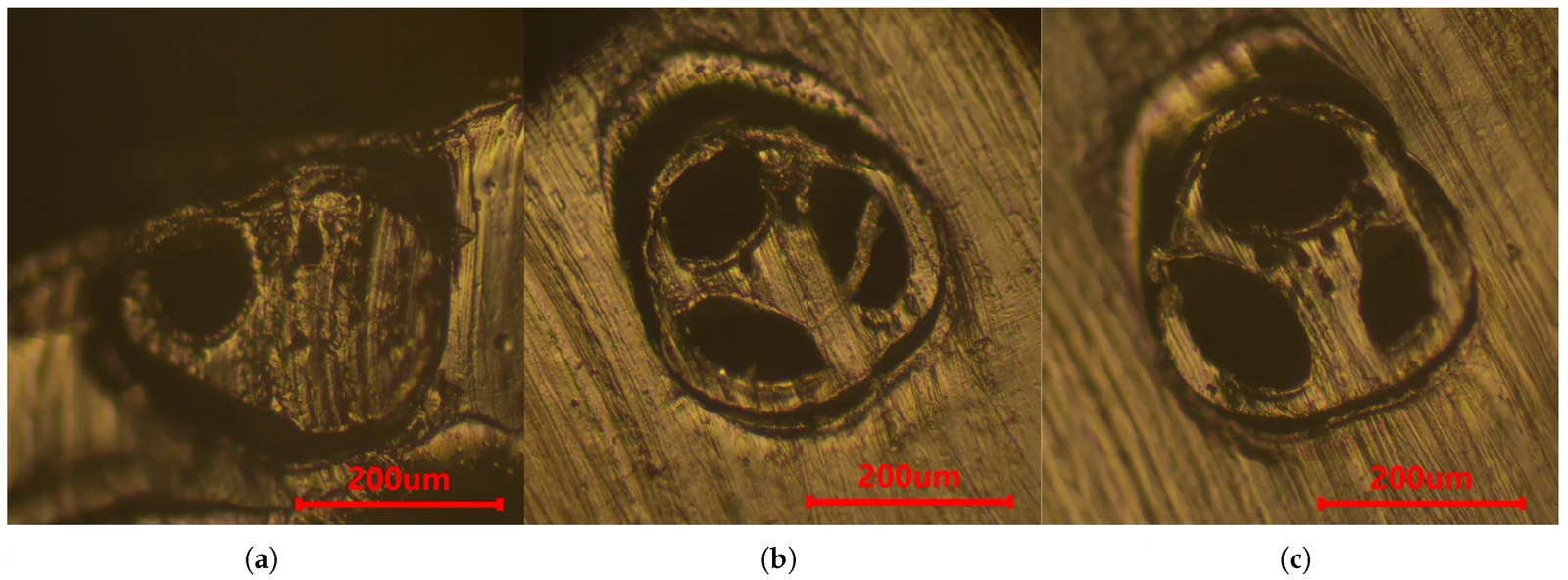
Microscopic images of drawn fiber cross-sections of PA6 three-channel preforms. Panels (**a**–**c**) show the cross-sections of replicates 1, 2, and 3 of the 5th (0.7) AR, respectively.

Statistical analysis provided no clear evidence to identify the most suitable AR(s) within the investigated range. Therefore, channel integrity was evaluated qualitatively using optical microscopy images of the cross-sections, captured all along the length of the drawn fibers for each AR. A minimum of five cross-sectional images were taken for each replicate. A representative image for each replicate is shown in Figure 13, Figure 14, Figure 15, Figure 16 and Figure 17.

Based on the optical microscope images of the cross-sections, the 2nd (0.4) and 3rd (0.5) ARs were the only configurations that continuously maintained the integrity of all three channels across all replicates.

## 4. Discussion

While previous studies have presented contrary findings regarding the optimal printing layer thickness [21,23], none of these investigations implemented PA6-based preforms for thermal drawing experiments. We hypothesize that the consistency observed in our layer thickness experiments may be driven by characteristics unique to PA6. Specifically, its semi-crystalline structure, sharp phase transitions, and hygroscopic nature likely contribute to the contrasting behavior concerning printing layer thickness.

Our first hypothesis is based on the consideration that polymer crystallinity could have impacted the inter-layer adhesion in 3D-printed preforms [38]. To achieve strong inter-layer adhesion during 3D printing, the newly deposited layer must impart enough heat to melt the surface of the underlying layer, to allow the polymer chains to entangle across the layer boundary. Amorphous polymers, such as polycarbonate (PC) and polyethylene terephthalate glycol (PETG), possess wider processing windows [39]. Consequently, a newly printed layer will hold enough thermal mass to melt the underlying layer, ensuring robust adhesion between the layers, with minimal dependence on printing layer thickness. In contrast, a semi-crystalline material with a well-defined melting temperature features a narrower processing window [40], making inter-layer adhesion highly sensitive to printing layer thickness. We propose that a 0.3 mm layer carries a larger thermal mass in comparison to 0.1 mm and 0.2 mm layers, allowing it to remain at ideal adhesion temperatures for a longer duration before solidifying. This extended period facilitates deeper polymer chain entanglement between layers, allowing a preform printed with a layer thickness of 0.3 mm to behave more like a homogeneous solid cylinder inside the furnace during the drawing process. Conversely, preforms printed at 0.1 mm or 0.2 mm are prone to melting unevenly, resulting in inconsistent fiber diameters. Previous attempts to study the impact of printing layer thickness focused on amorphous polymers such as PC [21] and PETG [23], with results suggesting that lower printing layer thicknesses produce more consistent fiber diameters. However, PA6 is a semi-crystalline thermoplastic, with a sharp melting transition [41]. Therefore, we hypothesize that implementing a larger layer thickness to print PA6 preforms enhances inter-layer adhesion during 3D printing, ultimately yielding more consistent fiber diameters during the thermal drawing process. While our ongoing experiments focus primarily on PA6, this hypothesis remains to be fully validated. To test its broader applicability, we plan to verify the crystallinity of the PA6 used in our experiments via Differential Scanning Calorimetry (DSC). Furthermore, we expect to conduct comparable layer thickness experiments with polyoxymethylene (POM) [42], thermoplastic polyurethane (TPU) [43], PC [21], and PETG [23]. POM is a semi-crystalline material that exhibits comparable thermodynamic behavior to PA6 during 3D printing and thermal drawing [42]. TPU is a semi-crystalline block co-polymer with a wide processing window [44]. PC and PETG are amorphous materials with verified thermal drawability [21,23]. Comparing these materials will allow us to further verify the combined impact of crystallinity and layer thickness on the consistency of drawn fiber diameters.

Moreover, we hypothesize that the moisture absorption and thermal shrinkage [45] during the printing process may have impacted the structural quality of preforms. PA6 is highly hygroscopic, and therefore it can absorb a significant amount of moisture while being printed [31]. A preform printed at either 0.1 mm or 0.2 mm layer thickness contains three times and one-and-a-half times as many physical layers, respectively, as one printed at a 0.3 mm layer thickness. Consequently, printing preforms at 0.1 mm and 0.2 mm resolutions takes comparatively longer. We speculate that these extended printing durations, and the higher number of physical layers in 0.1 mm and 0.2 mm preforms, increase the likelihood of moisture accumulation. Simultaneously, a higher number of physical layers introduces more repeated heating and cooling cycles during printing, intensifying thermal shrinkage and internal stresses within the preform [45]. During the thermal drawing process, furnace temperatures exceeding 100 °C convert any trapped moisture into steam, creating off-colored streaking [46] (commonly known as splay) in the fiber along the draw direction. Built-up thermal stresses would create bubble-like structures i.e., vacuum voids due to thermal shrinkage. These defects within and on the surface of the stretched preform can potentially lead to significant fluctuations in drawn fiber diameter. While moisture-related effects can be minimized by placing the preforms in a dehydrator prior to drawing, inherent thermal shrinkage cannot be entirely eliminated. Therefore, we speculate that the faster printing times and fewer physical layers of the 0.3 mm replicates minimized moisture exposure windows and layer-by-layer cooling dynamics, ultimately leading to more consistent fiber diameters during the drawing process.

**Figure 18:**
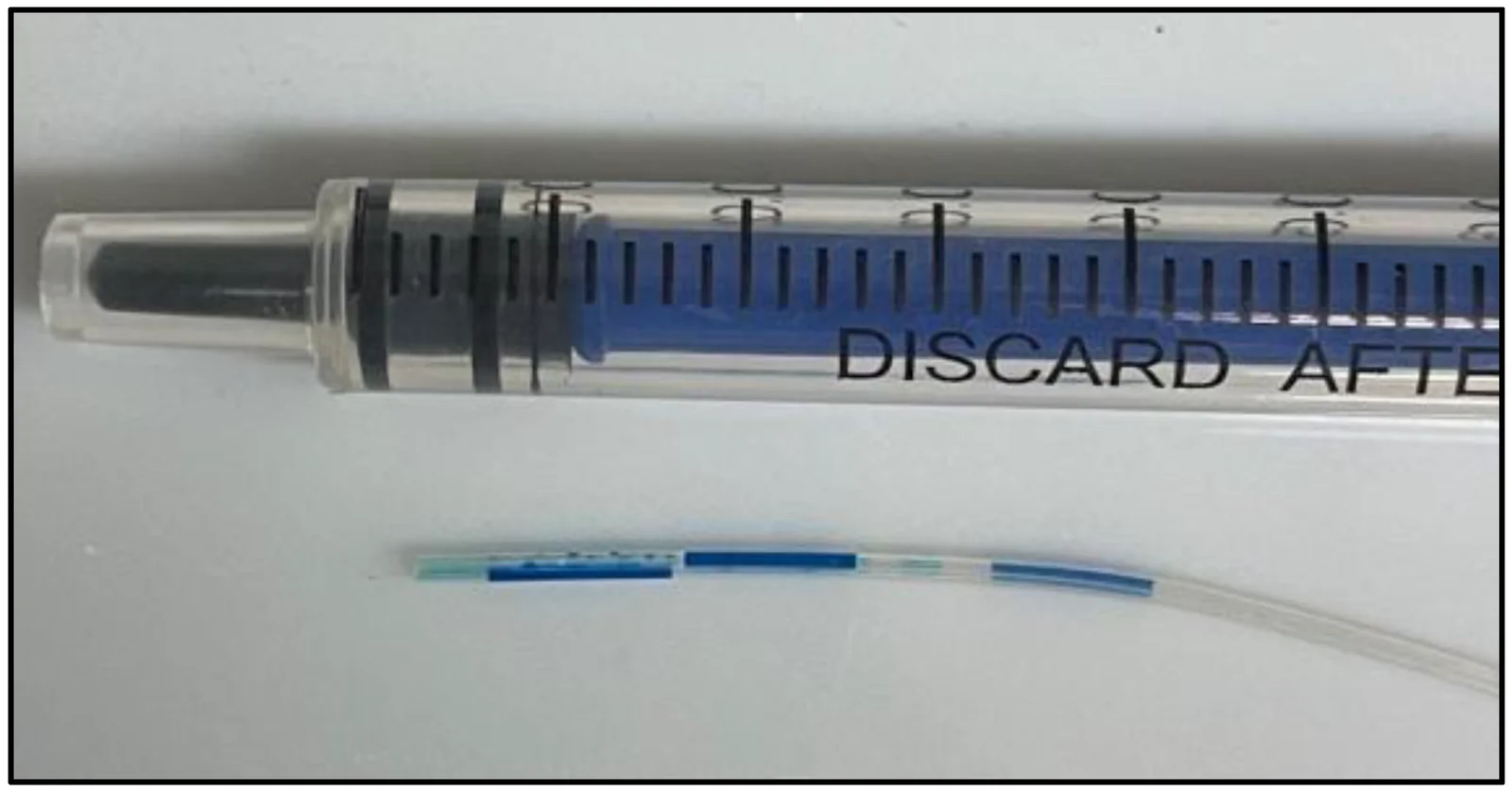
Side profile of PA6 three-channel preform. Blue food coloring has been sucked into each channel. This section of the fiber was intentionally drawn to a diameter of approximately 1000 μm to facilitate internal fluid suction to illustrate that the full length of the draw fiber maintained open channels.

From AR experiments, the 2nd (0.4) and 3rd (0.5) ARs yielded drawn fibers that continuously maintained internal three-channel architecture across all replicates (Figure 18). While all tested ARs yielded statistically indistinguishable fiber length dimensions, the channeled architecture collapsed in the 1st, 4th, and 5th ARs.

According to results from established models, the fiber length dimension is governed by the balance between the polymer viscosity and the draw tension [33], whereas the channel integrity depends on the balance between the inward pressure exerted by surface tension and outward pressure created by the pressurized trapped air inside each channel and structural resistance [47,48]. In the 1st AR, the internal channel diameter is relatively small in comparison to the outer diameter of a single channel. Therefore, the inward pressure exerted by the surface tension, which acts to minimize the surface area (i.e., radially inwards), overwhelms outward resistive pressure (i.e., radially outwards) exerted by the channel’s small volume [47,48]. In contrast, for the 4th and 5th ARs, the thin walls lack sufficient thermal mass, causing them to melt faster relatively. This sudden drop in viscosity prevents the melting preform from providing enough resistance against the draw tension [33], ultimately leading to a collapse of the channels. These results highlight that the 2nd and 3rd ARs achieve a vital hydrodynamic equilibrium between the competing forces, preserving both the consistency of the diameter and the integrity of the internal channels.

While we successfully obtained consistent fiber diameters for solid cylindrical preforms and consistent length dimensions for three-channel preforms, we encountered several limitations that led to several discrepancies during the 3D printing and the thermal drawing processes.

As detailed in Section 2.1, all PA6 preforms were printed using a custom-made filament. Therefore we ran into several failures while printing due to the inconsistencies in the diameter of this non-commercial filament, limiting the number of viable replicates for each experimental geometry. Furthermore, due to significant structural shaking at greater heights during printing, printable preform length is currently restricted to 15 cm. This causes a single thermal drawing experimental cycle to be restricted to a duration of 1500 s to 1800 s. To address this limitation, we are currently designing interlocking, puzzle-like structures for either end of the preforms, which will allow multiple preforms to be joined to extend the durations of the experiments.

**Figure 19:**
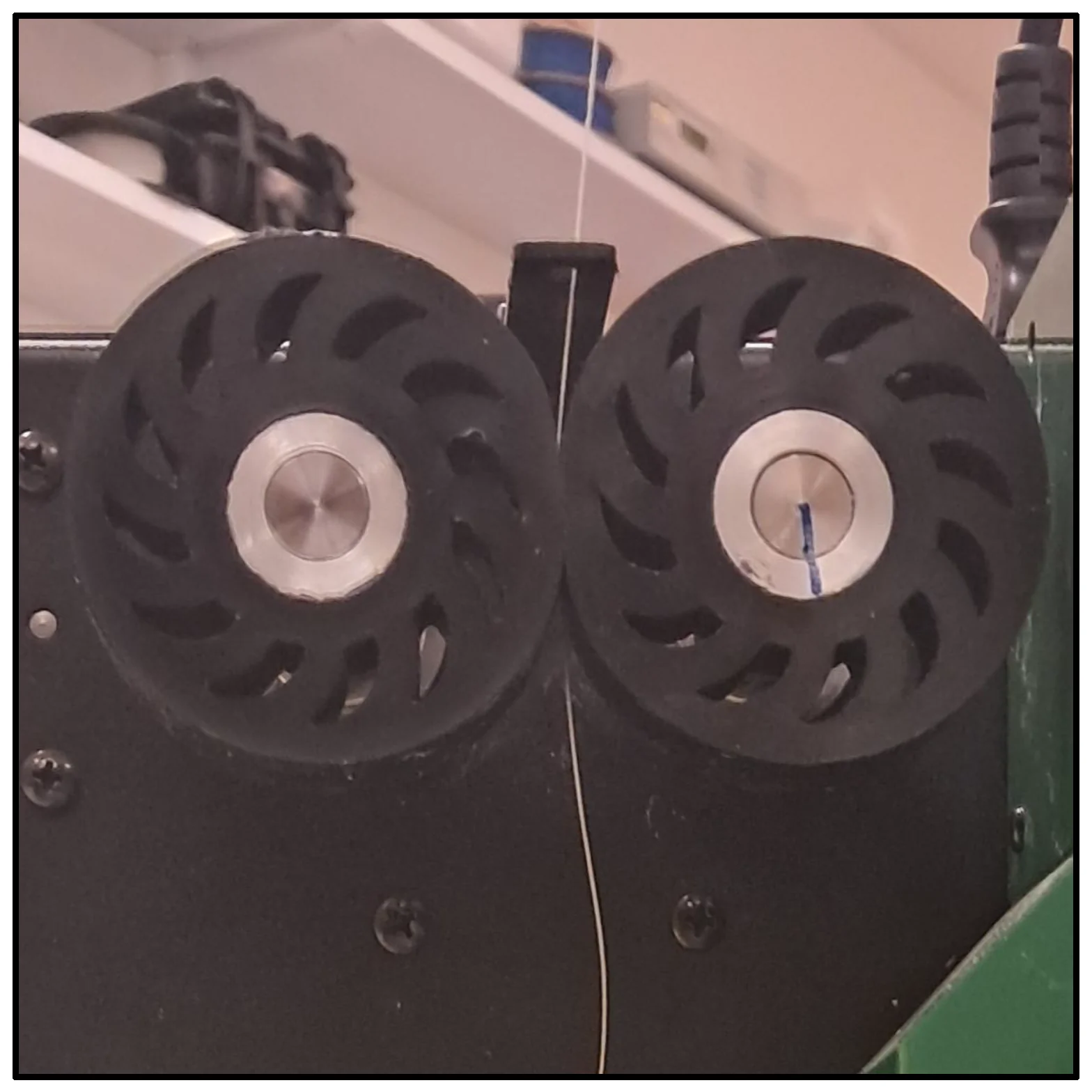
Side profile of the drive wheels of the winding mechanism.

During the AR experiments, while the 2nd and 3rd ARs continuously maintained open channels, the drawn fiber cross-sections did not perfectly resemble scaled-down versions of the original preform cross-sections. We attribute this to three primary limitations in our draw tower setup. First, while previous studies have achieved high-resolution, complex micro-scale cross-sections by implementing furnaces with three separate heating zones [21], our current draw tower only features two furnaces. We speculate that the addition of a third heating zone with a controlled temperature gradient will allow the neck-down region to gradually reach room temperature, likely improving the cross-sectional resolution of drawn fibers. Second, we hypothesize that the winding mechanism deforms the cross-sections of our fibers. As shown in Figure 19, for micro-scale dimensions, the drive wheels in the winder can easily crush and deform the fiber geometry. Considering that the verticality of our draw tower is restricted by the laboratory dimensions, the distance from the furnace to the drive wheels is only about 0.7 m. This distance may not provide sufficient time for the fiber to adequately cool down to room temperature before being spooled, increasing the susceptibility of the fiber to mechanically deform during the winding process. Finally, we speculate that the current slicing method used for microscopic imaging could have introduced additional geometric artifacts and deformations to imaged cross-sections. We utilized the blade from an ultrasonic cutter to slice the cross-sections, which could have inadvertently deformed the geometries during sample preparation. Given the above limitations, our current evaluation of internal channels and fiber cross-sections remains strictly qualitative.

Additionally, our current draw tower setup lacks the ability to actively control and monitor the pressure inside the preform channels and within the furnace. In our experiments, the three-channel preforms were sealed, trapping atmospheric air inside. As the preforms underwent preheating at 85 °C for 10 min followed by heating at 270 °C in the melting/softening zone, the internal channel pressure increased due to thermal expansion. However, during our experiments, we were unable to regulate the pressure inside the channels quantitatively. These unmonitored fluctuations may have influenced the homogeneity of cross-sectional geometries in our drawn fibers. To better preserve these geometries, future iterations of our setup will require active pressure regulation. In fact, recent thermal drawing studies have successfully implemented pressure as a regulatory parameter to optimize the cross-sectional consistency [49,50]. Such measures have been applied in the fabrication of advanced multilumen medical catheters [51]. Therefore, integrating systematic pressure control within our draw tower setup will be critical in future experiments for maintaining homogeneity in complex cross-sections.

The current draw tower setup utilizes a single-axis laser micrometer which only allows us to measure the length dimension in a single direction. While this would be sufficient to monitor the diameter of a circular cross-section, evaluating the length dimension of a complex, non-circular cross-section requires the assumption that the preform’s orientation remains fixed and consistent during the heating, softening, and the fiber drawing processes. To prevent measuring inaccuracies in future experiments, a multi-axis sensing setup should be implemented [21].

In this study, we successfully implemented thermal drawing to produce micro-scale fibers using 3D-printed PA6 preforms. These findings establish a foundation for fabricating monofilament fibers with superior mechanical properties using semi-crystalline polymer preforms. With further refinement, we believe these fibers may have the potential to be implemented in actuator and sensor applications for smart textiles and fabrics.

## 5. Conclusions

A series of thermal drawing experiments were conducted using 3D-printed PA6 preforms with two distinct cross-sectional geometries. Solid cylindrical preforms were utilized to determine the best-performing printing layer thickness for PA6 preforms among the tested values, while three-channel preforms were used to identify the most suitable AR(s) between the inner channel diameter and the outer diameter of a single channel. For semi-crystalline PA6, the highest printing layer thickness out of the tested values of 0.3 mm produced the most consistent fiber diameters over extended durations. These findings contradicted the conventional result on amorphous polymers, which concluded that a lower layer thickness yielded greater consistency [21]. We hypothesize that this deviation is inherent to the material properties of semi-crystalline PA6. Furthermore, while the statistical analysis of the length dimension measurements was unable to definitively establish an optimal aspect ratio, the qualitative evaluation of the internal channel integrity and general dimensional trends indicated that the 2nd (0.4) and 3rd (0.5) ARs produced the most promising results within the investigated range. We hypothesize that these ARs achieve a critical hydrodynamic equilibrium among the forces acting on the PA6 preform during the thermal drawing process, corroborating with theoretical and experimental observations from previous studies [33,47,48].

Although this study focused primarily on optimizing the design rules and process parameters for manufacturing complex monofilament fibers, the unique structural properties of resulting PA6 fibers have the potential to be utilized in a wide array of functional applications. Recently, structured monofilament fibers have been successfully integrated into soft robotics for both sensing [52] and actuating [53] applications. We believe that our fibers may have the potential to be integrated in such systems either directly or after undergoing post-processing. For instance, applying techniques such as cold-drawing [54] can enhance the performance of these fibers, potentially allowing them to serve as a platform in the next generation of wearable sensors [55] and actuator fibers [56]. Furthermore, given the superior mechanical and structural properties of PA6, the micro-scale channeled architecture holds potential for advanced future applications such as radiative cooling, where preserving hollow-channel integrity is critical for maximizing the internal surface area [57].

## Figures and Tables

**Table 1:** Process parameters and their corresponding values for 3D printing PA6 preforms.

Parameter	Value
Filament diameter	1.55 mm
Flow rate	21 mm^3^ s^−1^
Line width	0.4 mm
Infill density	100%
Infill pattern	Concentric
Printing temperature	270 °C
Print bed temperature	55 °C
Nozzle diameter	0.4 mm
Printing speed	80 mm s^−1^
Printing orientation	Vertical
Cooling fan	off

**Table 2:** Values of ARs used for printing the three-channel preforms.

Trial	Inner Channel Diameter (mm)	Outer Diameter (mm)	Aspect Ratio (AR)
1st	2.85	9.5	0.3
2nd	3.80	9.5	0.4
3rd	4.75	9.5	0.5
4th	5.70	9.5	0.6
5th	6.65	9.5	0.7

**Table 3:** Values of individual statistical metrics that were used to evaluate the best-performing layer thickness among the tested values for printing PA6 solid cylindrical preforms.

Layer Thickness (mm)	Replicate	Mean Diameter ($\bar{\mu }$) (μm)	Standard Deviation (SD) (μm)	Root Mean Squared Error (RMSE) (μm)	Coefficient of Variation (CV) (%)
0.1	R1 *	428	42	50	9.81
0.1	R2	420	59	62	14.05
0.1	R3	415	56	58	13.49
0.1	R4	427	56	62	13.11
0.2	R1	411	45	46	10.95
0.2	R2	416	69	71	16.59
0.2	R3	408	57	58	13.97
0.2	R4	416	64	66	15.38
0.3	R1	413	37	39	8.96
0.3	R2	402	35	35	8.71
0.3	R3	411	41	42	9.98
0.3	R4	422	38	44	9.00

* The time duration used to calculate the metrics for R1 of 0.1 mm layer thickness was restricted to 700 s to 1440 s.

**Table 4:** Mean values of statistical metrics among replicates for each layer thickness and values from the ANOVA analysis.

Layer Thickness (mm)	Mean CV Among Replicates (%)	Mean RMSE Among Replicates (μm)
0.1	12.62	58
0.2	14.22	60
0.3	9.16	40
ANOVA F-statistic	8.131	8.892
ANOVA *p*-value	0.010 *	0.007 *

* A *p*-value smaller than 0.05 indicates a significant difference in performance between the three layer thicknesses.

**Table 5:** Results from the post hoc independent-samples *t*-tests.

Layer Thickness Combinations	*p*-Value from the *t*-Test for CV	*p*-Value from the *t*-Test for RMSE
0.1 mm & 0.2 mm	0.340	0.731
0.1 mm & 0.3 mm	0.031 *	0.003 *
0.2 mm & 0.3 mm	0.022 *	0.028 *

* A *p*-value smaller than 0.05 indicates a significant difference in performance between the pair of layer thicknesses.

**Table 6:** Values of statistical metrics that were used to evaluate the most suitable AR within the investigated range for printing PA6 three-channel preforms.

Aspect Ratio (AR)	Replicate	Mean Diameter ($\bar{\mu }$) (μm)	Standard Deviation (SD) (μm)	Root Mean Squared Error (RMSE) (μm)	Coefficient of Variation (CV) (%)
1st (0.3)	R1	368	49	53	13.32
1st (0.3)	R2	377	38	48	10.08
1st (0.3)	R3	356	29	30	8.15
2nd (0.4)	R1	389	39	54	10.13
2nd (0.4)	R2	373	28	38	7.51
2nd (0.4)	R3	396	31	58	7.83
3rd (0.5)	R1	389	43	59	11.05
3rd (0.5)	R2	375	31	42	8.27
3rd (0.5)	R3	362	29	33	8.01
4th (0.6)	R1	368	55	59	14.95
4th (0.6)	R2	308	41	57	13.31
4th (0.6)	R3	370	35	42	9.46
5th (0.7)	R1	310	46	59	14.84
5th (0.7)	R2	341	53	53	15.5
5th (0.7)	R3	350	45	45	12.86

**Table 7:** Mean values of statistical metrics among replicates for each AR and values from the ANOVA analysis.

Aspect Ratio (AR)	Mean CV Among Replicates (%)	Mean RMSE Among Replicates (μm)
1st (0.3)	10.52	44
2nd (0.4)	8.49	50
3rd (0.5)	9.11	45
4th (0.6)	12.57	53
5th (0.7)	14.41	52
ANOVA F-statistic	4.210	0.477
ANOVA *p*-value	0.030 *	0.753

* A *p*-value smaller than 0.05 indicates a significant difference in performance between five ARs.

**Table 8:** Results from the post hoc Holm-adjusted pairwise comparisons.

Aspect Ratio (AR) Combinations	Holm-Adjusted *p*-Value for CV
1st (0.3) & 2nd (0.4)	1.000
1st (0.3) & 3rd (0.5)	1.000
1st (0.3) & 4th (0.6)	1.000
1st (0.3) & 5th (0.7)	0.844
2nd (0.4) & 3rd (0.5)	1.000
2nd (0.4) & 4th (0.6)	0.844
2nd (0.4) & 5th (0.7)	0.068
3rd (0.5) & 4th (0.6)	0.946
3rd (0.5) & 5th (0.7)	0.132
4th (0.6) & 5th (0.7)	0.100

## Data Availability

The experimental datasets generated during this study are available from the corresponding author upon reasonable request.

## Abbreviations

The following abbreviations are used in this manuscript:

PA6	Polyamide-6
FDM	Fused Deposition Modeling
AR	Aspect Ratio
PLA	Polylactic Acid
PE	Polyethylene
PS	Polystyrene
EGaIn	Gallium–Indium Alloy
GPs	Graphene Nanoplates
PU	Polyurethane
CAD	Computer-Aided Design
SD	Standard Deviation
CV	Coefficient of Variation
RMSE	Root Mean Square Error
ANOVA	Analysis of Variance
PC	Polycarbonate
PETG	Polyethylene Terephthalate Glycol
DSC	Differential Scanning Calorimetry
POM	Polyoxymethylene
TPU	Thermoplastic Polyurethane
